# Potential impacts of acupuncture on motor function recovery after ischemic stroke: insights from basic and clinical studies

**DOI:** 10.3389/fncel.2025.1623535

**Published:** 2025-08-13

**Authors:** Jia-Ling He, Liang-Xiao Ma, Jing-Si Wen, Yu-Xin Zhuang, Xu Qian, Ling-Hui Ma, Jing-Yun Xiu, Xiu-Yan Wang, Meng-Yu Chen

**Affiliations:** ^1^School of Acupuncture-Moxibustion and Tuina, Beijing University of Chinese Medicine, Beijing, China; ^2^The Key Unit of State Administration of Traditional Chinese Medicine, Evaluation of Characteristic Acupuncture Therapy, Beijing, China

**Keywords:** ischemic stroke, pathogenesis, motor function, rehabilitation, acupuncture

## Abstract

Ischemic stroke is one of the leading causes of death and long-term disability worldwide. A significant proportion of stroke survivors experience persistent motor impairments, which severely affect their quality of life and cause heavy social and economic burdens. Acupuncture has increasingly gained attention due to its remarkable efficacy in promoting motor function recovery after stroke, and it has been progressively endorsed as a post-stroke treatment option by clinical guidelines of numerous countries, despite its underlying mechanism is not yet fully understood. This review systematically evaluates existing basic and clinical studies to explore the potential mechanisms of acupuncture’s effects on motor function recovery after ischemic stroke and the optimal clinical strategies. Emerging evidence demonstrates that acupuncture-mediated post-stroke motor recovery is primarily attributed to its roles in restoring energy metabolism, inhibiting neuroinflammation, preventing neuronal apoptosis, promoting neuronal repair and regeneration, and regulating neuronal excitability. Additionally, individualized acupuncture modality involving syndrome-based selection of acupoints and stimulating methods is crucial for better rehabilitation outcome. Our findings elucidate the multidimensional impacts of acupuncture on motor function restoration following ischemic stroke, furnishing robust evidence and theoretical foundation for its clinical application.

## 1 Introduction

Stroke is a cerebrovascular disease that usually leads to localized damage to the central nervous system due to either blocked blood supply to the brain (ischemic stroke) or cerebral hemorrhage (hemorrhagic stroke), with ischemic stroke accounting for 76% of all cases (Virani et al., 2021). According to the latest global health statistics, stroke remains the second leading cause of death worldwide and is a major factor leading to permanent disability (GBD 2019 Stroke Collaborators, 2021). As the population ages and lifestyle changes, the incidence of stroke continues to rise, which has become a major challenge to global public health. More than two-thirds of stroke patients continue to experience varying degrees of motor function impairment after the acute phase (Handley et al., 2009; Wissel et al., 2013), which undermines their ability to live independently, severely impacts their quality of life and subsequently increases social and economic burdens (Gong et al., 2022). Although some patients achieve partial motor recovery through neural remodeling and compensation, those with severe injuries often evolve into permanent disability (Dimyan and Cohen, 2011). Consequently, motor function recovery is the primary focus of post-stroke rehabilitation.

Due to the complex manifestations of motor impairments after ischemic stroke, no clear management strategies have been established. Drug therapy is a common treatment, for example, baclofen and botulinum toxin for hypertonia, while haloperidol and diazepam are employed to control tremor and hemichorea-hemiballism (Creamer et al., 2018; Ristic et al., 2002; Gracies et al., 2015). Yet, such efficacy is generally confined to transient alleviation of symptoms. Stroke survivors often require long-term medication, which may lead to drug dependence and resistance, as well as a range of adverse reactions, including potential toxicity to liver and kidney (Falcone et al., 2024). Rehabilitation also represents a central strategy, such as the combination of task-specific training and general aerobic exercise (Dimyan and Cohen, 2011; Nudo et al., 1996; Taub et al., 2002). Despite this established approach, its overall effectiveness remains limited, as 15%–30% of patients continue to experience permanent disability even after intensive training and sustained physical activity (Lloyd-Jones et al., 2009). With advances in technology, particularly the support of nanotechnology, some new therapies have been proposed. Particularly the neural stem cell (NSC) therapy and exogenous material-based replacement therapy have shown preliminary preclinical success in promoting neural tissue regeneration (Lindvall and Kokaia, 2010; Zhong et al., 2010; Waris et al., 2022; Lee et al., 2017). In addition, controlling neural prosthetics through brain-computer interfaces offers a new pathway, which bypasses the damaged neural pathways and thereby becomes a training tool to promote the remodeling and functional recovery of the nervous system (Daly and Wolpaw, 2008). Although these new therapies show great potential, they are still in the stage of small-scale research and have not yet been widely applied. Furthermore, these therapies are associated with high costs and technical challenges, which hinder their widespread adoption in clinical treatment.

Acupuncture has been widely employed for the management of stroke in China for several millennia, particularly in the restoration of limb motor function. The World Health Organization has recommended acupuncture as a complementary and alternative therapy for stroke sequelae (World Health Organization, 2002). Meanwhile, it has been progressively endorsed as a post-stroke treatment option by clinical guidelines of numerous countries (Birch and Robinson, 2022). Unlike drug therapy, acupuncture both alleviates individual symptoms and fundamentally promotes nerve repair and improves motor function through multi-target and multi-channel mechanisms, such as repairing the damaged neural network, and restoring the function of neural circuits (Li et al., 2024; Mu et al., 2023). Hence, acupuncture exhibits significant potential for both research exploration and clinical application.

This review focuses on motor function recovery after ischemic stroke and provides a comprehensive evaluation of existing basic and clinical studies on acupuncture. Basic studies are examined to illustrate the mechanism of acupuncture, providing a theoretical basis for its clinical application. In parallel, clinical evidence is evaluated to assess and compare efficacy of different acupuncture protocols, with the aim of providing more precise guidance for clinical practice.

## 2 Methods

We performed a comprehensive literature search in PubMed, Web of Science, and Embase, covering publications from the inception of each database up to the present time. The search was limited to studies published in English and focused on ischemic stroke. The following keywords were used in various combinations: acupuncture, electroacupuncture (EA), stroke, cerebral infarction, motor dysfunction, motor impairment, movement disorder and rehabilitation. Following a thorough assessment, the information furnished in the following studies has been elucidated and discussed in detail.

## 3 Overview of motor impairments after ischemic stroke

### 3.1 Neurophysiological modulation of motor function

The neural modulation of motor activities is a complex and precise process that relies on the cooperation of multiple components of the central nervous system. The motor cortex, located in the frontal lobe, is the origin of voluntary movement and is responsible for issuing motor commands and regulating movement (Ebbesen and Brecht, 2017). The motor cortex does not directly innervate muscles, instead, it regulates movement through complex neural pathways. Layer 5 pyramidal neurons in the primary motor cortex send projections via the corticospinal and corticobulbar tracts to the interneurons in the spinal cord and brainstem, which then precisely regulate movement by activating or inhibiting lower motor neuron activity (Lemon, 2008; Grinevich et al., 2005; O’Donoghue et al., 1987). Additionally, the motor cortex connects multiple cortical and subcortical structures through neural pathways, including the somatosensory cortex, basal ganglia, motor thalamus, brainstem, and cerebellum, to finely regulate motion (Kinnischtzke et al., 2014; Osten and Margrie, 2013). Meanwhile, the motor cortex receives input from the primary somatosensory cortex to optimize motor commands by integrating sensory information (Petrof et al., 2015; Ferezou et al., 2007).

The basal ganglia, located deep within the white matter of the brain, primarily regulate the timing and intensity of movement to ensure coordination and fluidity (Yttri and Dudman, 2016). The striatum is the largest input nucleus of the basal ganglia which receives signals from the frontal lobe, sensory and motor cortices, and related thalamic regions (Mcgeorge and Faull, 1989; Gremel and Costa, 2013; Li et al., 2015). It selects the most appropriate behavior after integrating internal states, environmental information, and exercise plans (Klaus et al., 2019). The striatum includes direct medium spiny neurons (dMSNs) and indirect medium spiny neurons (iMSNs). Sustained activation of dMSNs increases motion, whereas sustained activation of iMSNs decreases motion (Kravitz et al., 2010). By controlling the activities of these two types of neurons, the cortex flexibly regulates the initiation, inhibition, frequency, and intensity of movement to meet different demands (Gurney et al., 2015; Yttri and Dudman, 2016).

The cerebellum delicately regulates movement primarily through feedback circles with other brain regions to maintain the accuracy and stability of action (Kim et al., 2024). The cerebellum is crucial for motor control, coordination, and learning, with its diverse regions affecting movement through specific pathways (Morton and Bastian, 2004). The medial cerebellar region receives and integrates inputs from the spinal cord, brainstem, and vestibular system to regulate key motor pathways, such as the vestibulospinal and reticulospinal tracts, in order to maintain postural balance and trunk stability (Ilg et al., 2008; Matsushita and Okado, 1981). The middle cerebellar region receives inputs from the cortex, spinal cord, and reticular nucleus, and projects signals to the red nucleus and cortex after integrating motor information, thereby coordinating movement (Asanuma et al., 1983b; Asanuma et al., 1983a). The lateral cerebellar region receives dense projections from cortical regions and sends signals to the red nucleus and cortex, primarily controlling the walk to ensure the consistency and rhythmicity of movement (Dum and Strick, 2003).

The brainstem integrates motor control signals from brain and spinal cord, and directly regulates the spinal cord circuitry, thereby controlling the initiation, speed, halt, and direction of movement (Leiras et al., 2022). As a central hub for regulating motor initiation and gait, the midbrain locomotor region (MLR) receives inputs from the cerebral cortex, basal ganglia, and brainstem sensorimotor regulatory regions to coordinate autonomous exploratory behavior and escape responses by conveying motor signals to the spinal cord via the reticulospinal tract (Caggiano et al., 2018; Dautan et al., 2021). Thus, the precise modulation of spinal motor circuits is attained via the synergistic actions of the excitatory medial tract and the inhibitory lateral tract (Brownstone and Chopek, 2018).

As the final executive link in motor control, the spinal cord receives and integrates descending signals from the central nervous system and peripheral sensory feedback to coordinate and execute reflex and voluntary and rhythmic movements (Nielsen, 2016). The anterior horn is the convergence of motor neurons, which is responsible for transmitting motor commands to the surrounding muscles (Negro and Farina, 2011). The cortex regulates motor neurons in the anterior horn through descending neural pathways, such as the corticospinal tracts and reticulospinal tracts, so as to ensure timely and coordinated muscle activity, thereby optimizing movement and maintaining postural stability (Teka et al., 2017; Menon and Vucic, 2021). In addition, a large number of spinal interneurons, distributed in the gray matter of the spinal cord, constitute a complex motor regulation network (Côté et al., 2018). Spinal interneurons continuously receive peripheral sensory information from the spinal cord dorsal horn and integrate it with descending signals from higher centers to flexibly balance the excitability and inhibition of the anterior horn, further regulating movement.

### 3.2 Motor impairments after ischemic stroke

#### 3.2.1 Neural structural damage

Although the brain accounts for only 2% of body weight, its energy demand accounts for 20% of the body’s total energy consumption (Sifat et al., 2022). Disruption of energy metabolism is a pathological feature of ischemic stroke (Yatsu et al., 1975). After ischemic stroke, brain tissue surrounding the occluded vessels becomes ischemic, and the blood flow in the core ischemic region is reduced by more than 80% (Back et al., 2004). This causes neurons to be damaged due to a sudden drop in energy supply (Lyden et al., 2019). Research has shown that among hemiplegic patients with hand dyskinesia after stroke, the ipsilateral thalamus displays severe metabolic inhibition, and thalamic metabolic activity correlates with the degree of motor function recovery, revealing the critical role of energy metabolism restoration in motor rehabilitation (Binkofski et al., 1996). Adenosine Triphosphate (ATP) exhaustion triggers ischemic cascade reactions, including failure of membrane ion pumps, cellular edema, and membrane depolarization (Lee et al., 2000; Hofmeijer and Van Putten, 2012). Neurons cannot maintain their normal transmembrane ion gradients, which triggers a series of pathophysiological processes, including excitotoxicity, mitochondrial dysfunction, oxidative and nitrative stress, neuroinflammation, protein misfolding, and apoptosis. These pathological mechanisms form a vicious cycle, ultimately leading to cell death (He Z. et al., 2020).

Chemokines, reactive oxygen species, and other factors produced by the ischemic cascade reaction trigger immune responses in the nervous system (Larrea et al., 2023). Persistent inflammation expands the extent of brain injury and severely impacts motor function after stroke (Larrea et al., 2023; Lukacova et al., 2021). Neuroinflammation directly damages local tissues in the early stages. Moreover, it promotes glial scar formation and inhibits neuronal regeneration, leading to long-term neuronal damage (Nishimura et al., 2007; Beck and Yaari, 2008). This further impairs motor function and eventually leads to chronic and persistent disability (Larrea et al., 2023; Min et al., 2012). Research confirms that excessive microglial activation after stroke significantly worsens motor function damage, which suggests that relieving neuroinflammation is crucial for recovering motor function after stroke (Lartey et al., 2014).

Disruption of energy metabolism and subsequent initiation of inflammation together lead to cellular dysfunction and apoptosis (Zhou et al., 2021; Pascotini et al., 2015). Extensive apoptosis occurs in the motor cortex, basal ganglia, and other motor control-related regions, causing disruption of the structure and function of motor circuits and ultimately leads to motor impairments. One study shows that early motor rehabilitation after ischemic stroke can protect neurons and promote the recovery of coordinated forelimb motor function by inhibiting neuronal apoptosis in middle cerebral artery occlusion (MCAO) rats (Zhang et al., 2013).

#### 3.2.2 Motor impairment

A complete neural structure is essential for the proper functioning of nerves in regulating motor activities. Given that the neural regulation of movement is a complex and precise network, injury to any component may impair motor function. After ischemic stroke, ischemic injury affects several brain regions involved in movement, leading to various motor impairments. The motor cortex exhibits distinct temporal characteristics following injury. During the acute phase, the main manifestations are muscle weakness, reduced and slowed movement. In the chronic phase, spasticity, clonus, and hypertonia occur due to the weakened inhibition of the cortex on the lower motor centers (Schieber and Poliakov, 1998; Laplane et al., 1977). The basal ganglia inhibits lower motor centers through glutamatergic and dopaminergic inputs, thereby preventing involuntary movements (Grillner et al., 2020), thus its injury primarily leads to contralateral hyperkinetic movement disorders, including dystonia, chorea, and tremor (Park, 2016). Moreover, the white matter tissue near the basal ganglia, the internal capsule, is frequently infarcted after ischemic stroke, leading to severe motor and sensory dysfunction in the contralateral limb (Horie et al., 2019). In contrast, ischemic injury in the cerebellum and brainstem is relatively rare. In over 90% of strokes, the cerebellum and brainstem structures involved in gait control remain intact (Beyaert et al., 2015). Although the spinal cord is not directly damaged after ischemic stroke, it is highly dependent on the regulation from higher centers. After ischemic stroke, the descending inhibitory signals to the spinal cord are weakened due to higher central nervous system injury, which leads to abnormal spinal excitability, increased muscle tone, and spasticity (Urbin et al., 2021; Segal, 2018).

After ischemic stroke, the nervous system initiates a spontaneous repair process to compensate for impaired motor function through limited functional recovery and compensation (Joy and Carmichael, 2021). Neuroplasticity constitutes the pivotal mechanism driving motor recovery after ischemic stroke. Through structural and functional remodeling, it reconstructs and regulates the damaged motor network to adapt to new motor requirements (Alia et al., 2017; Dimyan and Cohen, 2011). Patients often adopt new movement strategies and action patterns to replace pre-stroke movement behaviors, thereby compensating for motor function deficits (Bernhardt et al., 2017). The new motor mode often results in incomplete compensation, reduced precision, and abnormal movement patterns, which may limit motor recovery and even worsen motor impairment (Wahl et al., 2017; Whishaw, 2000). Therefore, timely and effective interventions are crucial. They promote the recovery of impaired function and prevents the spontaneous compensatory process from forming abnormal movement patterns, thereby maximizing overall motor function recovery.

## 4 Basic studies on acupuncture in promoting motor function recovery after ischemic stroke

Ischemic stroke causes extensive neuronal damage in the early stages, further hindering nerve repair and functional recovery. It disrupts the integrity of the neural network and weakens the regulatory capacity of the motor control system. Acupuncture may exert multidimensional modulation on such pathological changes and neuroplastic processes. It can improve energy metabolism, reduce inflammation, and inhibit apoptosis, thereby reducing neuronal injury and protecting the remaining neurons. Acupuncture also promotes neural plasticity, including enhancing axonal regeneration and synaptic remodeling, and regulating neuronal excitability to optimize the function of the motor circuit. Such roles of acupuncture enable its neuroprotection during the acute phase, while facilitating nerve repair and functional remodeling during the recovery phase, which offers crucial intervention strategies for motor function recovery after ischemic stroke (Table 1).

**Table 1:** Characteristic of basic studies.

Authors	Animal model	Acupoint(s)	Acupuncture method	Course of acupuncture	Stroke phase of study	Motor function behavioral testing indicator(s)	Molecular biology indicator(s)
Wu et al. (2017)	MCAO rat (I/R)	ST36, LI11	EA, 2/20 Hz, 30 min/day, once a day	7 days	Acute phase	MAS, CatWalk XT Gait Analysis, Rota-rod test	Glycolysis rate↑, p-AMPK*α*/t-AMPK*α* ratio↑
Lu et al. (2015)	MCAO, rat	PC6, LI11	EA, 2/15 Hz, 1 mA, 20 min/day, once a day	7 days	Acute phase	Longa Neurological Score	Lactate concentration↑, MCT1↑
Tian et al. (2022)	MCAO rat (I/R)	GV20, GV26	EA Pretreatment, 2/50 Hz, 30 min/day, once a day	5 days	Acute phase	Longa Neurological Score	MMP↑, LC3-II/LC3-I ratio↓, p-ULK1↓, FUNDC1↓
Nie et al. (2024)	MCAO, rat	GV20, ST36	EA, 2 Hz, 1 mA, 30 min/day, once a day	14 days	Early subacute phase	Beam-Balance Test	HMGB1↓, JNK↓, p-JNK↓
Liu et al. (2016b)	MCAO rat (I/R)	ST36, LI11	EA, 1/20 Hz, 4 V, 30 min/day, once a day	3 days	Acute phase	Longa Neurological Score	NF-*κ*B nuclear translocation↓, NF-*κ*B p65-positive cell count↓
Han et al. (2015)	MCAO, rat	PC6, LI11, SP8	EA, 2/15 Hz, 1 mA, 30 min/day, once a day	5 days	Acute phase	Longa Neurological Score	TNF-*α*↓, IL-1*β*↓, IL-6↓, TLR4↓, HMGB1↓, TRAF6↓, IKK*β*↓, NF-*κ*B p65↓
Lan et al. (2013)	MCAO rat (I/R)	ST36, LI11	EA, 1/20 Hz	once	Hyper-acute phase	Longa Neurological Score	TLR4↓, NF-*κ*B p65↓, p-I*κ*B↓, NF-*κ*B nuclear translocation↓, TNF-*α*↓, IL-1*β*↓, IL-6↓
Zhang et al. (2023a)	MCAO rat, (I/R)	GV14, GV9, GV4, GV20, BL17, BL18, BL23	MA	24 h, 36 h, 48 h, 72 h after MCAO(I/R)	Hyper-acute phase, acute phase	Longa Neurological Score	TGF-*β*↑, TNF-*α*↓, IL-1*β*↓, BIRC3 mRNA↓, LTBR mRNA↓, PLCG2 mRNA↓, TLR4 mRNA↓, TRADD mRNA↓
Liu et al. (2016a)	MCAO rat (I/R)	ST36, LI11	EA, 1/20 Hz, 6 V, 0.2 mA, 30 min/day, once a day	3 days	Acute phase	mNSS, CatWalk XT Gait Analysis	TNF-*α*↓, IL-1*β*↓, IL-6↓, NF-*κ*B nuclear translocation↓, NF-*κ*B p65↓
Ren et al. (2024)	MCAO mice (I/R)	GV20, GV26	EA, 4/20 Hz, 1 V-3 V, 1 mA–3 mA, 20 min/day, once a day	3 days	Acute phase	Rotarod Test, neurological deficit score	IL-6↓, TNF-*α*↓, IL-1*β*↓, CCL-2↓, CD206↓
Yao et al. (2023)	MCAO rat (I/R)	LU5, LI4, ST36, SP6	EA, 5 Hz, 2 mA, 20 min/day	3, 7 days	Acute phase	Longa Neurological Score, Grip Strength Test	STAT6↑, p-STAT6/STAT6 ratio↑, PPAR*γ*↑, p-PPAR*γ*↑, IL-10↑, TGF-*β*↑, M2 microglia↑, p-NF-*κ*B p65↓, M1 microglia count↓, IL-6↓, TNF-*α*↓
Wang et al. (2023a)	MCAO rat (I/R)	GV20	EA, 2/15 Hz, 1 mA, 20 min/day, once a day	3 days	Acute phase	Longa Neurological Score	IL-10↑, Treg cells↑, TNF-*α*↓, IL-1*β*↓, CXCL1 mRNA↓, CXCL2 mRNA↓, IL-17A↓
Zhang et al. (2023b)	MCAO, rat	MS5, MS6	MA, 30 min/day, once a day	14 days	Early subacute phase	Longa Neurological Score, Screen-Grabbing Test, Beam-Walking Test	p-IRE1↓, p-PERK↓, ATF6↓, CHOP↓, p-JNK↓, Caspase-3↓, Caspase-9↓
Xing et al. (2018a)	MCAO rat (I/R)	LI11, ST36	EA, 4/20 Hz, 4 V, 30 min/day, once a day	3 days	Acute phase	Longa Neurological Score	Bcl-2↑, p-Akt↑, p-PDK1↑, p-GSK-3*β*↑, Caspase-3↓, Bim↓, p-PTEN↓
Xing et al. (2018b)	MCAO rat (I/R)	LI11, ST36	EA, 4/20 Hz, 6 V, 1 mA, 30 min/day, once a day	3 days	Acute phase	Longa Neurological Score	Bcl-2-positive cells↑, Caspase-3-positive cells↓, Bim-positive cells↓, p-ERK1/2↓, p-JNK↓, p-p38↓
Kim et al. (2018)	MCAO mice (I/R)	GV20, GV14	EA, 2 Hz, 2 V, 20 min/day, once a day	12 days	Early subacute phase	Corner Test, Cylinder Test	BDNF↑, NT-4↑, VEGF↑, p-TrkB↑, p-CREB↑
Wang et al. (2021a)	MCAO rat (I/R)	GV20, ST36	EA,100 Hz, 1 mA, 30 min/day, once a day	14 days	Early subacute phase	Rotarod Test, Beam-Balance Test	BDNF↑, NGF↑, VEGF↑, Nogo-A↓, p75NTR↓
Deng et al. (2016)	MCAO rat (I/R)	GV20	EA, 2/10 Hz, 1 mA–2 mA, 30 min/day, 5 sessions/week	7, 14, 21, 28 days	Acute phase, early subacute phase	mNSS, Rotarod Test, Grip Strength Test	BDA-positive CST axon count↑, NF-200↑, GAP-43↑, RhoA↓, PriB↓
Kim et al. (2013)	Photothrombosis stroke (PTS) mice	GV20, GV14	EA Pretreatment, 2 Hz, 1 mA, 20 min/day, once a day	3 days	Hyper-acute phase, acute phase	Longa Neurological Score, Wire Hanging Test, Corner Test, Cylinder Test	SDF-1*α*↑, BDNF↑
Kim et al. (2014)	MCAO rat (I/R)	GV20, GV14	EA, 2 Hz, 2 V, 20 min/day, once a day	30 days	Early subacute phase	Rotation Device Test	BDNF↑, VEGF mRNA↑, p-PI3K/BrdU double-positive cell count↑
Xie et al. (2019)	MCAO rat (I/R)	GV20, GV24	EA, 1/20 Hz, 0.2 mA, 30 min/day, once a day	14 days	Early subacute phase	Longa Neurological Score	PSD-95 positive cell count↑, SYN positive cell count↑, pyramidal neuron synapse count↑
Ren et al. (2008)	MCAO, rat	PC6, TE5, SP6, ST36	EA, 10 Hz, 1 mA, 30 min/day, 6 sessions/week	7, 14, 28 days	Acute phase, early subacute phase	Balance Beam Walking Test	Dendritic spine density↑, Ephrin-A5 mRNA↑
Sun et al. (2022)	MCAO, rat	GB34	MA, 30 min/day, once a day	7 days	Early subacute phase	Longa Neurological Score, MAS, Gait Analysis	GABA↑, KCC2↑, GABA_A*γ*2_↑
Mu et al., 2022	MCAO, rat	GB34	MA, 30 min/day, once a day	6 days	Early subacute phase	Longa Neurological Score, MAS, Gait Analysis, Foot Balance Test	GABA↑, KCC2↑, GABA_A*γ*2_↑
Wang et al. (2021c)	MCAO, rat	GB34	MA, 30 min/day, once a day	7 days	Early subacute phase	MAS, Screen Test	KCC2↑, GABA_A*γ*2_↑
Wang et al. (2020)	MCAO, rat	GB34	MA, 30 min/day, once a day	7 days	Early subacute phase	Longa Neurological Score, MAS	GABA↑, GABA-T↓

EA, Electroacupuncture; MA, Manual Acupuncture; ST36, Zusanli; LI11, Quchi; PC6, Neiguan; GV20, Baihui; GV26, Shuigou; SP8, Diji; GV14, Dazhui; GV9, Zhiyang; GV4, Mingmen; BL17, Geshu; BL18, Ganshu; BL23, Shenshu; LU5, Chize; LI4, Hegu; SP6, Sanyinjiao; MS5, Middle line of Vertex in scalp acupuncture; MS6, Anterior Oblique Line of Vertex-Temporal; GV24, Shenting; TE5, Waiguan; MAS, Modified Ashworth Scale; mNSS, Modified Neurological Severity Score.

### 4.1 Acupuncture reduces nerve damage to improve motor function

#### 4.1.1 Acupuncture regulates energy metabolism

Oxidative metabolism of glucose is the primary energy source for the brain, ensuring the survival and function of neurons (Zheng and Wang, 2018b). In cellular energy regulation, AMP activated protein kinase (AMPK) functions as a crucial energy sensor, detecting changes in cellular energy and regulating abnormal energy states. AMPK can be activated when energy decreases. Subsequently, it increases metabolism-related proteins expression and inhibits biosynthetic pathways to increase ATP (Hardie et al., 2012). After ischemia–reperfusion (I/R) injury, glucose metabolism in the affected hemisphere of rats is significantly lower than in the contralateral hemisphere, and EA can regulate this condition. Additionally, EA enhances energy production and reduces unnecessary energy consumption in brain tissue by activating AMPK, significantly improving gait and athletic ability in rats (Wu et al., 2017). In ischemia and hypoxia following ischemic stroke, due to inhibition of glucose oxidation metabolism, lactate can serve as an alternative energy substrate for neurons (Roumes et al., 2021; Bliss and Sapolsky, 2001). Monocarboxylate Transporter 1 (MCT1), widely distributed in rat brain tissue, promotes the unidirectional transport of monocarboxylates across the plasma membrane, including lactate and pyruvate (Vijay and Morris, 2014). EA upregulates MCT1 expression in astrocytes around the ischemic area and promotes the release of lactate produced by intracellular anaerobic fermentation into the extracellular space, which increases extracellular lactate concentration and provides energy substrates for injured neurons (Lu et al., 2015).

Mitochondria are central to cellular energy metabolism, and their dysfunction is considered a hallmark of I/R injury, making them a critical target for alleviating post-stroke motor impairments (Gibbs et al., 2016). Dysregulation of mitochondrial dynamics and quality control can lead to mitochondrial dysfunction, and even trigger mitochondrial autophagy (Wu et al., 2016). Unc-51-like kinase 1 (ULK1) plays a crucial role in the initial stages of mitochondrial autophagy (Ganley et al., 2009; Wirth et al., 2013). FUN14 domain containing 1 (FUNDC1) acts as a receptor for mitochondrial autophagy under hypoxia and is activated through phosphorylation at the Serine17 site mediated by ULK1. Upon activation, it binds to microtubule-associated protein light chain 3 (LC3) and links mitochondria and autophagosomes, promoting mitochondrial autophagy (Liu et al., 2012; Wu et al., 2014). This process is negatively regulated by the mammalian target of rapamycin (mTOR), a key modulator of cell growth. It prevents ULK1 activation by phosphorylating the Serine-757 site of ULK1, consequently inhibiting ULK1-mediated mitochondrial autophagy (Huang et al., 2011; Kim et al., 2011). EA pretreatment activates mTOR, downregulates p-ULK1, LC3-II/LC3-I, and FUNDC1 levels, which inhibits I/R-induced mitochondrial autophagy and restores mitochondrial membrane potential (MMP). This significantly reduces mitochondrial abnormalities, decreases the number of autolysosomes, which protects neurons from I/R damage and ultimately decreases longa neurological scores (Tian et al., 2022).

#### 4.1.2 Acupuncture alleviates neuroinflammation

After ischemic stroke, severe mitochondrial damage can trigger complex neuroinflammation, which further worsens neuronal injury and significantly impedes motor function recovery. The Toll-like receptor 4 (TLR4)/nuclear factor kappa B (NF-*κ*B) signaling pathway plays a particularly crucial role in acute inflammation. TLR4 is primarily responsible for recognizing damage-associated or pathogen-associated molecular patterns and initiates immune responses through binding the adaptor protein myeloid differentiation primary response 88 (MyD88) (Barton and Medzhitov, 2003; Stierschneider and Wiesner, 2023). High mobility group box 1 (HMGB1), a key nuclear protein and immune regulatory factor, is released from damaged neurons and glial cells into the extracellular space under ischemia and hypoxia (Wu et al., 2010). I/R injury promotes the rapid binding of HMGB1 to TLR4, which triggers the phosphorylation and degradation of I*κ*B and leads to the migration of the NF-*κ*B subunits (p65/p50) from the cytoplasm to the nucleus. Ultimately, NF-*κ*B activates the transcription of genes related to inflammation and immunity in the nucleus, thereby triggering and aggravating inflammation (Ridder and Schwaninger, 2009; Bhatt and Ghosh, 2014). TNF receptor-associated factor 6 (TRAF6), a downstream factor of TLR4, also participates in regulating the NF-*κ*B pathway (Song et al., 1997). It can phosphorylate I*κ*B by activating I*κ*B Kinase (IKK), thereby promoting the activity of the NF-*κ*B pathway (Wang et al., 2001; Deng et al., 2000). Additionally, TRAF6 further enhances NF-*κ*B activity by activating the c-Jun N-terminal kinase (JNK) signaling pathway, leading to sustained neuroinflammation (Darnay et al., 1999).

EA alleviates inflammation in striatal neurons of rats with cerebral ischemia by downregulating HMGB1, JNK, and p-JNK levels, thereby improving balance and motor coordination (Nie et al., 2024). Additionally, EA inhibits I*κ*B phosphorylation and NF-*κ*B p65 nuclear translocation by reducing TLR4 and its downstream factors, such as TRAF6, IKK*β*, tumor necrosis factor-alpha (TNF-*α*), interleukin-1*β* (IL-1*β*), and interleukin-6 (IL-6). This alleviates inflammatory damage in MCAO rats and improves neurological function (Liu et al., 2016b; Han et al., 2015; Lan et al., 2013). Further research shows that EA inhibits the NF-*κ*B pathway by downregulating the key genes expression related to NF-*κ*B, significantly reducing IL-1*β* and TNF-*α* levels and increasing tumor necrosis factor-*β* (TNF-*β*) levels. Ultimately, EA reduces edema, neuronal damage, and inflammatory infiltration in the ischemic core area caused by I/R and reduces longa neurological scores (Zhang X. et al., 2023).

Microglia are resident immune cells in the central nervous system, playing a key role in regulating immune responses, particularly in central nervous system disorders such as stroke, Parkinson’s disease, and Alzheimer’s disease (Hu et al., 2014; Keren-Shaul et al., 2017). Following activation of the TLR4/NF-*κ*B signaling pathway, microglia rapidly undergo activation and functional polarization. They tend to shift towards the pro-inflammatory M1 phenotype rather than the anti-inflammatory M2 phenotype. Subsequently, a series of pro-inflammatory cytokines are released, further worsening inflammation and expanding neuronal damage (Holtman et al., 2017; Shi et al., 2019). EA significantly inhibits excessive activation and proliferation of microglia in the sensory and motor cortex surrounding the infarction and prevents their polarization towards the M1 type, which reduces the expression of TNF-*α*, IL-1*β*, and IL-6 in both the cortex and serum. This alleviates I/R-induced neuroinflammation and improves motor coordination, balance, and gait in rats (Liu et al., 2016a; Ren et al., 2024). The Janus Kinase (JAK)/Signal Transducer and Activator of Transcription (STAT) pathway is a critical intracellular signaling pathway that binds to cytokines, hormones, and other molecules through receptors on the cell surface, transmits signals to the nucleus, and regulates gene transcription (Xin et al., 2020; Renauld, 2003). In the later stages of inflammation, anti-inflammatory factors such as interleukin-4 (IL-4) and interleukin-13 (IL-13) activate JAK1, which in turn activates STAT6. Together with peroxisome proliferator-activated receptor *γ* (PPAR*γ*), they promote microglia polarization towards the M2 type, ultimately fostering an anti-inflammatory response and tissue repair (He Y. et al., 2020a). EA increases the total expression of STAT6 and PPAR*γ* in microglia and promotes their activation, thereby facilitating the polarization of M1 microglia towards M2 and regulating the levels of corresponding pro-inflammatory and anti-inflammatory factors. This reduces longa neurological scores and improves muscle strength in the hind limbs of rats (Yao et al., 2023).

Th17 cells primarily participate in immune responses by secreting pro-inflammatory factors such as interleukin-17 (IL-17), interleukin-21 (IL-21) and interleukin-22 (IL-22) (Stockinger and Veldhoen, 2007). Treg cells primarily prevent excessive immune responses and autoimmune diseases by secreting immunosuppressive factors, such as transforming growth factor-beta (TGF-*β*) and interleukin-10 (IL-10). Under normal conditions, they inhibit overactive T helper 17 (Th17) cells and maintain immune tolerance and an anti-inflammatory response (Afzali et al., 2007; Liesz et al., 2009). The balance between Th17 cells and Treg cells is crucial in regulating neuroinflammation and restoring exercise capacity after stroke (Liu et al., 2015; Dolati et al., 2018). C-X-C motif chemokine ligand 1 (CXCL1) and C-X-C motif chemokine ligand 2 (CXCL2) are important inflammatory chemokines that promote Th17 cells differentiation and exacerbate neuroinflammation in combination with pro-inflammatory factors (Wojkowska et al., 2014). EA promotes the differentiation of Treg cells and IL-10 secretion in brain tissue, while downregulating the gene expression of CXCL1 and CXCL2, as well as the levels of interleukin-17A (IL-17A), TNF-*α*, and IL-1*β*. This ultimately reduces neuroinflammation and reduces longa neurological scores (Wang et al., 2023a, 2023b).

#### 4.1.3 Acupuncture inhibits cell apoptosis

Caspase-mediated apoptosis plays a critical role in neuronal death after ischemic stroke (Love, 2003). Caspases are a class of cysteine proteases, including both initiator and executioner types, that play a central role in cell apoptosis. Pro-apoptotic factors regulate caspase activation along with the anti-apoptotic factor B-cell lymphoma 2 (Bcl-2), such as Bcl-2 interacting mediator of cell death (Bim), Bcl-2 antagonist of cell death (Bad), and Bcl-2 associated x protein (Bax). Mitochondria damaged by ischemic stroke release cytochrome c, which binds to the apoptotic protease activating factor 1 (Apaf-1) and procaspase-9, forming apoptotic bodies and initiating a series of apoptotic events (Zhang and Armstrong, 2007; Love, 2003). Executioner caspases, primarily caspase-3, complete the final stages of apoptosis by degrading the genome and breaking down the cytoskeleton (Unnisa et al., 2023).

The endoplasmic reticulum (ER) is the primary organelle responsible for protein synthesis, transport, and the maintenance of intracellular Calcium ion (Ca^2+^) homeostasis. The imbalance in Ca^2+^ homeostasis caused by cerebral ischemia leads to the unfolded protein response (UPR) and accumulation, which in turn induces ER stress and initiates apoptosis (Han et al., 2021; Marciniak and Ron, 2006; Walter and Ron, 2011). Studies have shown that ER stress induced by cerebral ischemia is a key pathological mechanism related to damage to neurons, glial cells, and endothelial cells (Rissanen et al., 2006; Zhao et al., 2018; Haupt et al., 2020). Targeted inhibition of ER stress and the UPR can effectively alleviate experimental I/R injury (Zhao et al., 2018; Liu et al., 2020). The UPR, activated by ER stress, activates the expression of downstream pro-apoptotic factors by core sensors including inositol-requiring enzyme 1 (IRE1), protein kinase r -like endoplasmic reticulum kinase (PERK), and activating transcription factor 6 (ATF6) (Walter et al., 2018). Acupuncture reverses ischemia-induced ER swelling by downregulating the expression of p-IRE1, p-PERK, and ATF6. This inhibits the activity of pro-apoptotic factors such as JNK and C/EBP-homologous protein (CHOP), and downregulates the levels of caspase-9 and caspase-3, thereby inhibiting apoptosis of cortical penumbra neurons induced by ER stress and alleviating paralysis or spasticity after ischemic stroke (Zhang Y. et al., 2023).

The mitogen-activated protein kinase (MAPK) pathway is a crucial regulator of cell differentiation, inflammation, and apoptosis. It consists mainly of three functional branches: the extracellular signal-regulated kinase (ERK) pathway, JNK pathway, and p38 pathway. The dynamic balance between these pathways is crucial for determining cell survival or apoptosis (Xia et al., 1995; Peti and Page, 2013). Studies show that ERK1/2 is overexpressed in MCAO animals, and inhibiting ERK1/2 phosphorylation can reduce focal infarct volume and brain damage and provide neuroprotection (Zhang et al., 2010; Wang et al., 2003; Namura et al., 2001). JNK and p38 are important therapeutic targets in ischemic stroke, as they promote inflammatory responses, induce neuronal apoptosis, and exacerbate ischemic damage (Gao et al., 2005; Zheng et al., 2018a; Jiang et al., 2014; Barone et al., 2001). EA restores the balance of the ERK/JNK/p38 pathway by downregulating the activation of ERK1/2, JNK, and p38 in cortical infarcted areas. This promotes Bcl-2 expression and downregulates the levels of caspase-3 and Bim ultimately reducing longa neurological scores (Xing et al., 2018b).

Protein kinase B (Akt) is a key molecule that inhibits neuronal apoptosis (Vidal et al., 2022; Zheng et al., 2024; Liu et al., 2025). Phosphatidylinositol 3-kinase (PI3K) can induce the phosphorylation and activation of Akt. After activation, it recruits Akt and 3-phosphoinositide-dependent kinase 1 (PDK1) to the membrane by promoting the conversion of phosphatidylinositol 4,5-bisphosphate (PIP2) to phosphatidylinositol 3,4,5-trisphosphate (PIP3) (Alessi et al., 1996; Stokoe et al., 1997). PDK1 phosphorylates the Threonine 308 site of Akt, enabling it to regulate the activity of various substrates such as glycogen synthase kinase 3 beta (GSK3*β*), Bad, and Bim, thus playing an anti-apoptotic role (Vidal et al., 2022; Kaidanovich-Beilin and Woodgett, 2011; Datta et al., 1997; Qi et al., 2006). The phosphatase and tensin homolog (PTEN) located on chromosome 10 dephosphorylates the Threonine 308 site of Akt by catalyzing the conversion of PIP3 to PIP2, thereby inhibiting the anti-apoptotic effect of Akt (Maehama and Dixon, 1998; Li et al., 1997; Lee et al., 2004). EA upregulates the phosphorylation levels of PDK1, Akt, and GSK-3*β* in the cortex surrounding the infarction, inhibits PTEN expression, significantly reduces caspase-3 and Bim, and reverses the decrease in Bcl-2 induced by ischemia. This significantly reduces infarct volume and decreases the proportion of apoptotic cells, so as to reduce longa neurological scores in rats with cerebral ischemia (Xing et al., 2018a).

**Figure 1:**
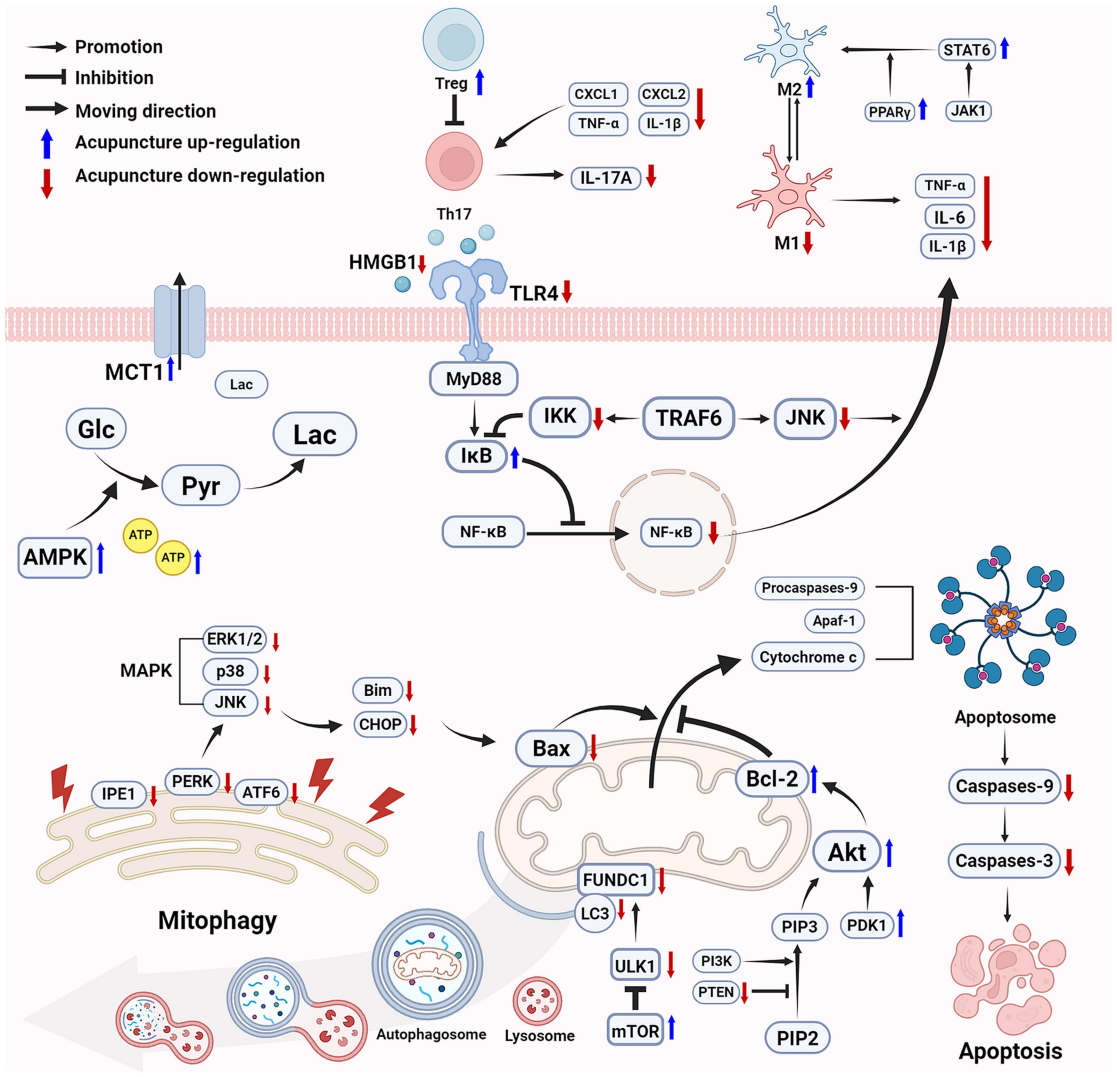
Acupuncture improves energy metabolism, reduces inflammation, and inhibits cell apoptosis following ischemic stroke to promote motor function recovery. Acupuncture effectively increases ATP levels and improves energy metabolism through multiple mechanisms. Acupuncture inhibits the NF-*κ*B pathway in acute inflammation, promotes the polarization of microglia from M1 to M2, and enhances Treg cells to inhibit Th17 cells while reduces IL-17A levels. Acupuncture protects mitochondrial function by activating the mTOR signaling pathway and inhibiting ULK1-mediated mitophagy, reduces ER stress, modulates the MAPK pathway, and activates the PI3K/Akt pathway to enhances the cellular anti-apoptotic capacity. Ultimately, acupuncture improves energy metabolism, alleviates inflammatory and inhibits cell apoptosis to provides neuroprotection, thereby promoting the recovery of motor function. (Created with biorender with permission to publish).

Taken together, the major mechanisms involved in the efficacy of acupuncture in promoting motor function following ischemic stroke via improving energy metabolism, reducing neuroinflammation, and inhibiting cell apoptosis, are shown in Figure 1.

### 4.2 Acupuncture restructures neural circuits to improve motor function

#### 4.2.1 Acupuncture facilitates nerve repair and regeneration

Neurotrophic factors, including brain-derived neurotrophic factor (BDNF), nerve growth factor (NGF), neurotrophin 3 (NT3), and neurotrophin 4 (NT4), participate in the development of the nervous system and the repair process following nerve injury by binding to specific receptors. BDNF and NT4 activate cAMP response element-binding protein (CREB) by binding to tropomyosin receptor kinase B (TrkB), upregulating genes related to nerve repair and growth, and promoting neuronal repair. NGF primarily exerts its neurotrophic effect by binding to tropomyosin receptor kinase A (TrkA) (Bai et al., 2019). Vascular endothelial growth factor (VEGF) is a key growth factor responsible for the generation and expansion of blood vessels. It provides neuroprotection and promotes nerve regeneration by inducing angiogenesis (Plate et al., 1999; Böcker-Meffert et al., 2002). EA increases the expression of BDNF, NT4, and VEGF, promotes the activation of TrkB and CREB, facilitates NSCs proliferation and differentiation, thereby alleviating striatal atrophy in MCAO/R mice and restores bilateral paw motor function. Its effect is stronger than that of mouse bone mesenchymal stem cells transplantation, particularly in terms of motor function related to ipsilateral turning (Kim et al., 2018).

After activation of the corresponding signaling pathways by neurotrophic factors, cytoskeletal remodeling is initiated, and the direction of axonal growth is guided by microtubules and microfilaments, thereby promoting the reconstruction of neural networks (Markus et al., 2002; Chen et al., 2017). Neurite outgrowth inhibitor A (Nogo-A) binds to the Nogo-66 receptor 1 (NgR1) and releases Ras homolog gene family member A (RhoA) in combination with the p75 neurotrophin receptor (p75NTR) (Schwab and Strittmatter, 2014). RhoA further activates Rho kinase (ROCK), in turn leading to actin cytoskeleton recombination, resulting in cone collapse and inhibition of neurite outgrowth (Fan et al., 2016). EA combined with constraint-induced exercise upregulates the levels of NGF, VEGF, and BDNF and inhibits the expression of Nogo-A and p75NTR, which significantly improves movement balance in MCAO/R rats (Wang D. et al., 2021). Growth-associated protein 43 (GAP-43) and neurofilament 200 (NF-200) promote axonal regeneration and synaptic plasticity, while paired immunoglobulin-like receptor B (PirB) inhibits neuronal burst growth by activating RhoA, thereby suppressing motor function recovery after ischemic stroke (Deng et al., 2018). EA upregulates the expression of NF-200 and GAP-43, while inhibiting PirB and RhoA expression to relieve the inhibition of axonal regeneration, which effectively repairs the motor pathway between the brain and spinal cord, ultimately enhancing muscle strength and promoting motor function recovery in rats (Deng et al., 2016). Postsynaptic density protein 95 (PSD-95) and synapsin (SYN) are critical proteins in synapses, playing a key role in regulating synaptic strength and activity-dependent synaptic plasticity (Béïque and Andrade, 2003; Tarsa and Goda, 2002). EA improves the decreased number and ultrastructure of synapses after I/R injury by increasing the number of PSD-95-positive and SYN-positive cells, thereby promoting neural plasticity in the brain (Xie et al., 2019). Ephrin-A5 participates in synapse formation and maturation by binding to EphA receptors (Otal et al., 2006). EA upregulates ephrin-A5 expression, increases the density and length of dendritic spines in the infarcted cortical area, thereby promoting functional recovery following ischemic stroke (Ren et al., 2008).

NSCs, as the primary source of neuronal regeneration, promote neural repair and motor function recovery by proliferating, differentiating, and migrating to generate new neurons, astrocytes, and oligodendrocytes (Tang et al., 2017). Stromal cell-derived factor 1 alpha (SDF-1*α*) promotes neural regeneration and behavioral recovery after ischemic stroke by enhancing the recruitment of endogenous NSCs (Luo et al., 2014; Deng et al., 2021; Zhao et al., 2015). Three days of EA pretreatment increase BDNF levels in the brain tissue of photothrombosis stroke mice and upregulates SDF-1*α* in plasma, significantly improving vestibular motor function, sensory motor function and forelimb symmetry (Kim et al., 2013). EA also increases the number of newly formed NSCs in the hippocampus, promotes their differentiation into neurons or astrocytes, and upregulates the levels of BDNF and VEGF (Kim et al., 2014).

#### 4.2.2 Acupuncture regulates neuronal excitability

Neuronal excitability refers to the ability of neurons to respond to stimuli and generate action potentials, directly affecting the normal function and stability of neural circuits (Turrigiano, 2011). After ischemic stroke, the connections between different regions of the nervous system related to movement are severely disrupted, causing an imbalance in neuronal excitability and motor impairments (Li et al., 2019; Hubli et al., 2012). Glutamate (Glu), the primary excitatory neurotransmitter in the central nervous system, maintains normal neuronal excitability by mediating the influx of Ca^2+^ (Hansen et al., 2021). Under pathological conditions, abnormal accumulation of excitatory amino acids in synaptic gaps can cause sustained neuronal overexcitation, leading to synaptic transmission disorders and Ca^2+^ overload. This disrupts neural network homeostasis and damages neural circuits related to motor control. Research shows that after ischemic stroke, impaired high-level central regulatory function leads motor neurons to frequently send abnormal nerve impulses, causing sustained muscle spasms and worsening motor impairments and disabilities (Trompetto et al., 2019). Gamma-aminobutyric acid (GABA) is the primary inhibitory neurotransmitter in the central nervous system, produced by the decarboxylation of Glu catalyzed by glutamate decarboxylase 67 (GAD67) and degraded by GABA-transaminase (GABA-T) (Lee et al., 2019). GABA inhibits neuronal excitability through two distinct pathways. Firstly, it diminished the excitatory signals of glutamatergic neurons and inhibits Glu release via presynaptic inhibition. Secondly, through postsynaptic inhibition, it binds to GABA receptors to promote Chloride ion (Cl^−^) influx, which subsequently leads to neuronal membrane hyperpolarization and a reduction in neuronal excitability (Chalifoux and Carter, 2010; Kaila, 1994; Li et al., 2002). The Potassium-Chloride co-transporter 2 (KCC2), located on the neuronal cell membrane, maintains low intracellular Cl^−^ levels by expelling Cl^−^, thereby facilitating GABA-mediated Cl^−^ influx and effectively inhibiting excessive excitability in motor neurons (Rivera et al., 2005; Watanabe et al., 2009). Several studies show that acupuncture upregulates GABA levels, enhances the expression of KCC2 and GABAA, and inhibits GABA-T activity in the nervous system of MCAO rats, thereby restoring normal neuronal excitability and promoting functional recovery of spastic limbs after ischemic stroke (Sun et al., 2022; Mu et al., 2022; Wang J. X. et al., 2021; Wang et al., 2020).

**Figure 2:**
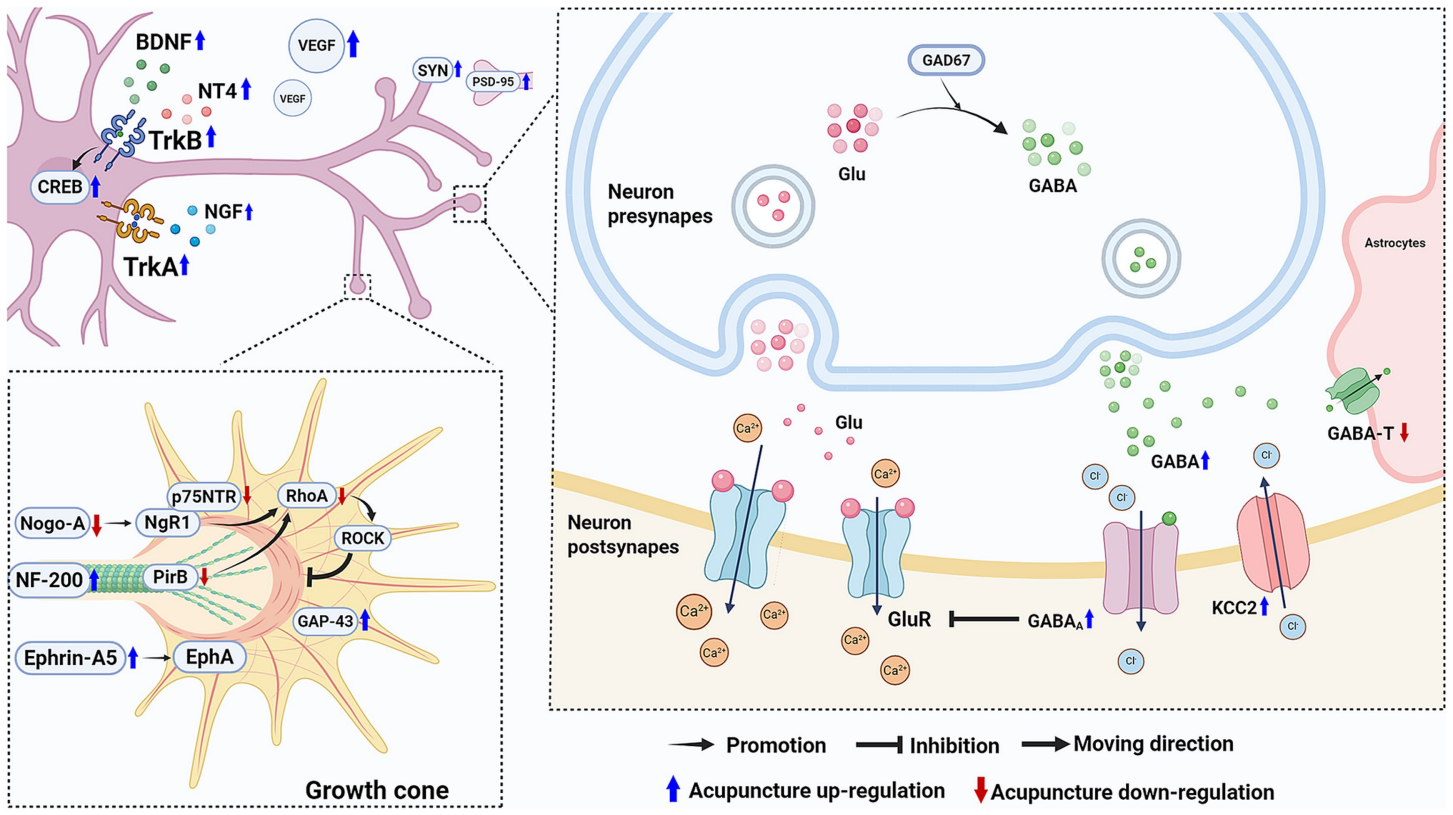
Acupuncture facilitates nerve repair and regeneration and regulates neuronal excitability following ischemic stroke to promote motor function recovery. Acupuncture upregulates neurotrophic factors and increases VEGF levels to support nerve repair. Besides, it promotes axon regeneration and downregulate Nogo-A and p75NTR to inhibit the Rho/ROCK pathway’s suppression of neurite growth. It also increases the expression of SYN and PSD-95 to enhance synaptic plasticity. Regarding neuronal excitability, acupuncture enhance GABA’s inhibitory effect on neuronal excitability, helping to restore normal neuronal excitability and the motor control functions of the nervous system. (Created with biorender with permission to publish).

The major mechanisms involved in the efficacy of acupuncture in improving motor function after ischemic stroke via facilitating nerve repair and regeneration and regulating neuronal excitability are shown in Figure 2.

## 5 Clinical studies on acupuncture in promoting motor function recovery after ischemic stroke

### 5.1 Outcome measures of acupuncture effects

Currently, several methods are used in clinical practice to comprehensively evaluate the efficacy of acupuncture in promoting post-stroke motor function recovery. The Fugl-Meyer Assessment (FMA) is the most commonly used scale for evaluating motor function, widely employed to objectively quantify motor, sensory, and joint function impairment in stroke patients (Fugl-Meyer et al., 1975). Nine studies used FMA to assess motor recovery in post-stroke patients (Wang et al., 2023a; Wayne et al., 2005; Xie et al., 2022; Tian et al., 2016; Gao et al., 2012; Xiong et al., 2020; Zhan et al., 2023; Bai et al., 2013; Wang et al., 2025). Motor and sensory impairment after ischemic stroke severely affects patients’ ability to perform daily activities. Therefore, the Barthel Index (BI) is often used to assess the ability to perform activities of daily living. It evaluates patients’ independence in basic daily activities, such as eating, dressing, and walking, and is used for rehabilitation assessment in stroke, Alzheimer’s disease, and spinal cord injury (Sulter et al., 1999). Eight studies utilized BI to evaluate functional independence in post-stroke patients (Wayne et al., 2005; Xie et al., 2022; Tian et al., 2016; Gao et al., 2012; Duc Nguyen et al., 2023; Zhan et al., 2023; Bai et al., 2013; Wang et al., 2025). The combined use of the FMA and BI comprehensively and dynamically evaluates the recovery status of patients.

Despite these scales are rich in content and convenient to use, they still have certain limitations. Scoring relies on the evaluator’s experience, introducing subjective bias, while limited sensitivity may reduce their effectiveness in detecting mild motor impairments. Combining subjective scales with objective indicators improves the accuracy and objectivity of evaluations. It provides a more comprehensive and accurate reflection of the effect of acupuncture on motor function recovery after ischemic stroke. Electromyography (EMG) effectively reveals weakened muscle strength, abnormal muscle tone, and motor control disorders caused by central nervous system injury in stroke patients by recording muscle electrophysiological activity. Two studies use EMG to evaluate muscle function after acupuncture (Duc Nguyen et al., 2023; Wang et al., 2025). Functional magnetic resonance imaging (fMRI) is a key technique for revealing the functional reorganization of the central nervous system after stroke. Three studies use fMRI to evaluate the effects of acupuncture on brain functional networks (Wang et al., 2023b; Schaechter et al., 2007; Zhan et al., 2023). They revealed the strength of brain network functional reorganization and spontaneous neural activity by analyzing functional connectivity and low-frequency amplitude. These imaging results reflect the activity and recovery of motor-related brain areas, highlighting the potential of acupuncture in promoting brain functional reorganization and enhancing neural plasticity. The use of other evaluation indicators is shown in Table 2.

**Table 2:** Characteristic of clinical studies.

Authors	Sample size	Acupuncture method	Course of acupuncture	Stroke phase of study	Outcome(s)
Wang et al. (2023b)	53	MA, 30 min/day, 5 sessions/week	2 weeks	Early subacute phase	FMA↑, fMRI
Wayne et al. (2005)	33	EA, 60 min/day, 2 sessions/week	10 weeks	Chronic phase	MAS↓, ROM↑, FMA↑, BI↑
Xie et al. (2022)	90	MA, 30 min/day, 5 sessions/week	4 weeks	Late subacute phase	FMA↑, BI↑, MMT↑
Tian et al. (2016)	68	EA, 5/20 Hz, 30 V, 1 mA–2 mA, 30 min/day, 6 sessions/week	2 weeks	Early subacute phase	NIHSS↓, FMA↑, BI↑
Gao et al. (2012)	106	MA, 45 min/day, once a day	4 weeks	Subacute phase	FMA↑, BI↑, NDS↓
Xiong et al. (2020)	72	MA, 3–4 h/day, 6 sessions/week	8 weeks	Late subacute phase	FMA↑, MMSE↑, LOTCA↑, ADL↓
Zhan et al. (2023)	108	MA. 30 min/day, 5 sessions/week	8 weeks	Subacute phase	FMA↑, BI↑, mRS↑, fMRI
Bai et al. (2013)	120	MA, 30 min/day, 6 sessions/week	4 weeks	Early subacute phase	FMA↑, BI↑
Wang et al. (2025)	90	TEAS, 20 Hz, 100 Hz, 30 min/day, 3 sessions/week	4 weeks	Late subacute phase, chronic phase	FMA↑, MAS↓, BI↑, EMG
Duc Nguyen et al. (2023)	120	EA, 50 Hz–100 Hz, 30 min/day, 5 sessions/week	6 weeks	Subacute phase	BI↑, MSG↑, mRS↓, EMG
Schaechter et al. (2007)	7	EA, 2 sessions/week	10 weeks	Chronic phase	fMRI

EA, Electroacupuncture; MA, Manual Acupuncture; TEAS, Transcutaneous Electrical Acupuncture Stimulation; ROM, Range of Motion; MMT, Manual Muscle Testing Scale; NIHSS, National Institutes of Health Stroke Scale; NDS, Neurological Deficit Score; MMSE, Mini-Mental State Examination; LOTCA, Loewenstein Occupational Therapy Cognitive Assessment; ADL, Activity of Daily Living; MSG, Muscle Strength Grading, mRS: Modified Rankin Scale.

### 5.2 Acupuncture intervention modalities

#### 5.2.1 Stimulation sites

**Figure 3:**
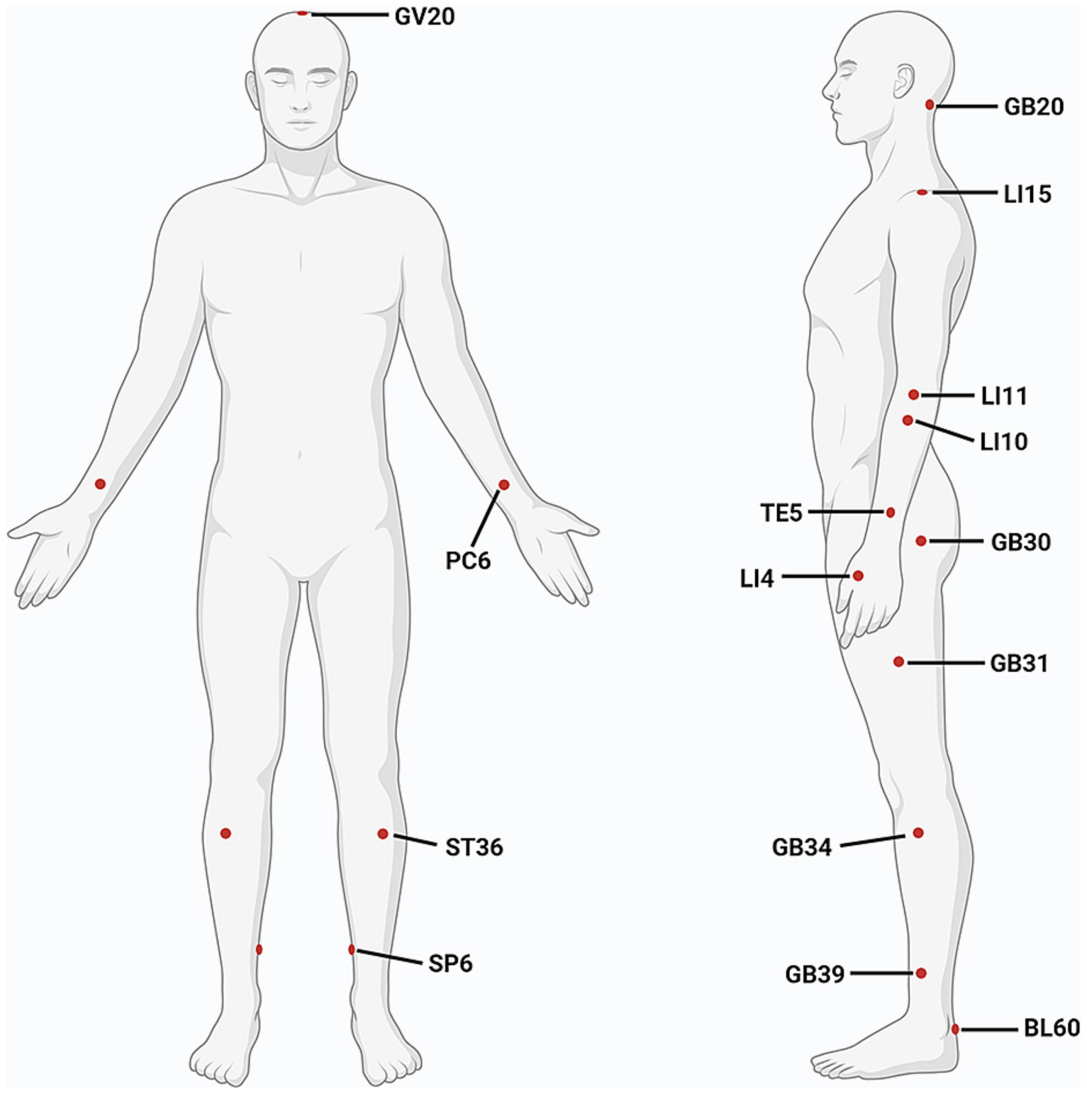
Commonly used acupoints for improving motor function after ischemic stroke. (Created with biorender with permission to publish).

It is a feature of acupuncture that appropriate acupoints are selected based on individual’s symptoms and syndromes (a series of clinical manifestations reflecting the pathogenesis of a disease). The choice of stimulation sites is an important factor affecting the efficacy of acupuncture. Considering that basic studies primarily focus on exploring the mechanisms of acupuncture, to optimize experimental controllability and reproducibility, a limited number of acupoints and simplified acupuncture techniques are typically used. The two most commonly used acupoints are GV20 and ST36. However, clinical studies place more emphasis on individualized treatments to achieve better effects, therefore, more acupoints are usually applied, such as GB34, LI4, GV20, LI15, LI11, SP6, and TE5. The appearance frequency of most commonly-used acupoints is shown in Table 3 and Figure 3.

**Table 3:** Utilization frequency of commonly used acupoints.

Basic experiments	Frequency (times)	Clinical trials	Frequency (times)
GV20-Baihui	10	GB34-Yanglingquan	7
ST36-Zusanli	9	LI4-Hegu	7
LI11-Quchi	8	TE5-Waiguan	4
GB34-Yanglingquan	4	LI15-Jianyu	4
GV26-Shuigou	3	LI11-Quchi	4
GV14-Dazhui	3	SP6-Sanyinjiao	3
PC6-Neiguan	2	GV20-Baihui	3
		GB20-Fengchi	3
		GB31-Fengshi	3
		GB30-Huantiao	3
		PC6-Neiguan	3
		LI10-Shousanli	3
		ST36-Zusanli	3

Conventional treatment typically targets the affected limb to facilitate motor function restoration. Nonetheless, acupuncture applied to the healthy limb also confers significant therapeutic benefits. Research shows that activity in the healthy hemisphere is increased during the first 10 days after stroke, followed by a gradual increase in activity in the impaired hemisphere. This dynamic neural activation process is closely linked to the recovery of motor function (Marshall et al., 2000; Ward et al., 2003). When the lesion affects most of the motor-related areas, the role of the healthy hemisphere in functional reorganization and motor recovery is especially critical (Di Pino et al., 2014). One study compares the therapeutic effects of acupuncture on the healthy and affected sides. The results show that under the same acupoint selection, needling on the healthy limb has a more significant effect on improving FMA and BI scores, and reducing neurological deficit score (NDS) (Gao et al., 2012). This suggests that acupuncture on the healthy limb may promote overall motor function recovery by regulating the function of the healthy hemisphere. Its underlying mechanism requires further exploration.

#### 5.2.2 Stimulation methods

Existing research and classical theories suggest that different acupuncture techniques can significantly influence treatment efficacy (Davis et al., 2012; Wang J. et al., 2021). Compared to manual acupuncture, EA provides stable and continuous stimulation and accurately activates specific acupoints by adjusting pulse width, intensity, and frequency (Zhang et al., 2022). A study showed that EA is more effective than manual acupuncture in reducing National Institutes of Health Stroke Scale (NIHSS) scores and improving FMA and BI scores (Tian et al., 2016). Additionally, transcutaneous electrical acupuncture stimulation (TEAS) stimulates acupoints directly through the skin by attaching electrode pads. Combining TEAS, particularly in 100 Hz, with routine care significantly improves FMA and BI scores, increases limb co-contraction rates, and reduces MAS score and spastic muscle activity levels in patients with post-stroke spastic hemiplegia (Wang et al., 2025). Fire needle therapy is a method of rapidly penetrating the acupoint with a red burning needle tip to treat diseases. A meta-analysis indicates that fire needle performs better in reducing MAS than manual acupuncture especially in the upper limbs. In other scales, such as FMA, BI, and NDS, fire needle also shows a more significant effect (Qiu et al., 2021). By the way, warm needle acupuncture, which combines acupuncture and moxibustion, can deeply stimulate acupoints and enhance efficacy by transmitting warmth from burning moxa wool through the needle. A network meta-analysis compares the efficacy of various acupuncture techniques and finds that warm needle acupuncture is more effective in relieving spasticity in elderly stroke survivors, while manual acupuncture was more beneficial in improving overall motor function (Zhu G. C. et al., 2024). This suggests that the personalized selection of acupuncture techniques based on specific conditions is an effective strategy for improving clinical efficacy.

#### 5.2.3 Intervention time

The Stroke Recovery and Rehabilitation Roundtable (SRRR) classifies acute cerebral ischemia into five phases: hyperacute (within 24 h), acute (1–7 days), early subacute (7 days to 3 months), late subacute (3–6 months), and chronic (over 6 months) (Bernhardt et al., 2017). Most stroke survivors undergo spontaneous functional recovery in the early stages, but the duration varies depending on the affected neurological system (Cramer et al., 2007). For example, motor function typically improves within weeks to months after stroke, while language function recovery may take months to years (Nakayama et al., 1994). The first week to the first month after stroke is a critical period for neural plasticity, making this stage a key focus for rehabilitation therapy and clinical studies (Krakauer et al., 2012; Biernaskie et al., 2004). Although the optimal time window for acupuncture intervention remains undetermined, existing evidence indicates that earlier initiation and increased treatment frequency improve motor function and alleviate inflammatory responses (Wu et al., 2023; Xu et al., 2020). This may be linked to the mechanism of acupuncture that alleviates nerve damage during the acute phase of stroke by improving energy metabolism, regulating inflammation, and inhibiting cell apoptosis. A meta-analysis shows that early acupuncture intervention, particularly within 48 h after stroke, significantly improves FMA and BI scores, with efficacy lasting up to 15 days after onset, significantly better than late intervention (Zhuo et al., 2021). Nevertheless, current clinical studies primarily focus on the subacute and chronic phases, with relatively limited studies on the acute phase. Greater emphasis on early-stage acupuncture in future studies may help refine intervention timing and improve the efficacy of motor function recovery.

#### 5.2.4 Combined therapies

In clinical rehabilitation after ischemic stroke, a comprehensive intervention incorporating multiple treatment methods is commonly employed. Acupuncture can significantly enhance the effectiveness of motor function recovery when combined with conventional rehabilitation training, medication therapy, and other techniques. Several meta-analyses show that combining conventional rehabilitation, medication therapy, and mirror training with acupuncture further enhances motor function and accelerates the rehabilitation process (Cai et al., 2017; Lv et al., 2021; Peng et al., 2024; Tao et al., 2023; Zhan et al., 2018; Zhang et al., 2024; Zhu T. et al., 2024). Additionally, compared to using EA alone, a comprehensive plan that combines conventional rehabilitation therapy demonstrates superior performance in modulating the electromyographic frequency and amplitude in post-stroke patients with motor impairments. It also effectively enhances motor function and daily living ability (Duc Nguyen et al., 2023). These findings suggest that acupuncture, as an effective complementary therapy, is more beneficial when combined with conventional rehabilitation treatment than when used alone. In clinical practice, the cooperative effects of multiple intervention methods can optimize motor function recovery and significantly improve the quality of life. Current studies directly comparing the efficacy of acupuncture and conventional rehabilitation therapy remain limited. Future high-quality research evaluating their independent effects is needed to clarify the respective advantages of each approach and provide stronger evidence to support therapeutic strategies.

## 6 Challenges and recommendations for future studies

### 6.1 Advancements and limitations in basic studies

The exploration of the mechanisms by which acupuncture promotes motor function recovery following ischemic stroke offers a scientific foundation for its clinical application, and is of paramount importance for understanding such a traditional therapy and facilitating its wider clinical adoption in post-stroke rehabilitation. Acupuncture exerts neuroprotective and reparative effects through multiple pathways and targets, facilitating motor function recovery. During the acute injury phase after ischemia, acupuncture restores energy balance in neural tissue by promoting glycolysis and lactate metabolism, reducing mitochondrial damage, and regulating mitophagy. Neuroinflammation plays a critical role in early nerve damage and long-term motor dysfunction. Acupuncture effectively inhibits inflammation and promotes neuroprotection by suppressing excessive activation of the TLR4/NF-*κ*B signaling pathway, balancing microglial polarization, and restoring the Th17/Treg cell balance. It also inhibits neuronal apoptosis by regulating ER stress, the MAPK pathway, and the PI3K/Akt pathway. During the neural repair phase, acupuncture repairs damaged neural network structures by upregulating neurotrophic factors, promoting axonal growth and synaptic plasticity, and regulating the proliferation and differentiation of NSCs. Besides, acupuncture regulates neuronal excitability to ensure normal transmission of neural signals, providing the necessary foundation for the recovery of neural function. In summary, acupuncture provides neuroprotection by reducing ischemia-induced nerve damage in the early stages of ischemic stroke and promotes the reconstruction of the nervous system and repair of neural circuits in later stages, facilitating comprehensive motor function recovery across multiple stages.

Currently, basic studies on acupuncture mainly focus on regulating specific signaling pathways or repairing ischemic areas. However, the overall remodeling of neural networks, especially the repair of complex motor neural circuits after ischemic stroke, is critical in determining motor function recovery (George and Steinberg, 2015). The mechanism of acupuncture is multi-level and multi-dimensional, offering unique advantages in promoting the overall recovery of neural network structure and function, although many of its underlying mechanisms remain unexplored. Recent research has shown that using projection-specific and mononuclear RNA sequencing techniques to identify characteristic neurons associated with movement and observe their directed regeneration to natural target areas is essential for motor function recovery (Squair et al., 2023). Therefore, using modern technologies such as gene silencing or knockout, virus tracing, optogenetics, chemical genetics, small animal functional magnetic resonance imaging, two-photon microscopy, and combining single-cell sequencing and spatial transcriptomics, to deeply observe the repair and activity of neural circuits and explore how acupuncture promotes the functional reconstruction of motor-related brain regions and specific neural circuits has become a new research trend.

### 6.2 Suggestion for optimizing clinical studies

In addition to basic studies, clinical studies in this field may provide optimized acupuncture approaches for post-stroke motor dysfunction. Research indicates that at different stages of motor recovery, patients’ rehabilitation needs for neural functions vary. The effectiveness of acupuncture largely depends on the selection of stimulation sites and techniques (Stinear, 2010). Therefore, targeted acupuncture treatment should be used at the different stages of recovery to maximize rehabilitation effectiveness, which warrants further investigation. The optimal timing for acupuncture intervention remains unclear. However, multiple studies indicate that early intervention is critical for functional recovery after ischemic stroke, and early acupuncture treatment can significantly enhance motor function recovery (Coleman et al., 2017; Lou et al., 2024). Given that acupuncture can effectively inhibit nerve damage during the acute phase, initiating acupuncture treatment as early as possible may help promote motor function recovery. Furthermore, basic studies show that EA pretreatment can regulate mitochondrial autophagy, promote NSC proliferation and differentiation, thereby exerting neuroprotective and reparative effects, and improving motor function (Kim et al., 2013; Tian et al., 2022). This suggests that acupuncture both alleviates injuries after ischemic stroke and enhances the body’s tolerance to such injuries, indicating its potential preventive effects. Despite this, the clinical research and application of acupuncture pretreatment remain limited. Future research should explore the mechanisms and clinical effects of acupuncture pretreatment, and develop corresponding acupuncture pretreatment protocols for high-risk stroke populations.

Acupuncture has become an ideal choice for promoting motor function recovery when combined with other therapies, due to its non-invasive nature, simplicity, and good patient compliance. Combining acupuncture with medication, exercise rehabilitation, and other treatment methods can significantly enhance clinical efficacy. Currently, innovative technologies such as stem cell transplantation, brain-computer interfaces, robotic assistance, and non-invasive brain stimulation have been used to promote post-stroke motor function recovery but have not yet been integrated with acupuncture research (Raffin and Hummel, 2018; Soekadar et al., 2015; Muir et al., 2020; McCrary et al., 2020). Future research should investigate the combined effects of these innovative therapies and acupuncture, expand the application scenarios of acupuncture, and provide new strategies for improving motor function after ischemic stroke in clinical practice.

Although current clinical studies have demonstrated the positive effects of acupuncture in promoting motor function recovery, several methodological issues remain noteworthy. First, due to the inherent characteristics of acupuncture interventions, implementing conventional blinding methods presents certain challenges, which may affect the objectivity of study outcomes. Second, some studies included small sample sizes, resulting in insufficient statistical power and limited generalizability of the findings. In addition, many clinical studies lack standardized acupuncture protocols, with insufficiently detailed descriptions of intervention parameters. Future research should focus on designing more scientifically rigorous randomized controlled trials with appropriately calculated sample sizes. Moreover, it is recommended that researchers adhere strictly to the CONSORT statement and the STRICTA guidelines to ensure transparent and systematic reporting of both intervention details and study outcomes. These improvements will contribute to a more robust evidence base for the clinical application of acupuncture in motor function recovery following ischemic stroke.

### 6.3 Challenges from basic to clinical studies

Although basic studies have identified many potential targets and effective pathways in treatment and have reported significant therapeutic effects, they still face multiple challenges when translating research results to clinical practice due to differences between basic and clinical studies.

Firstly, experimental ischemic stroke is primarily modeled by creating permanent ischemia or reperfusion through the suture method, which simulates blood flow obstruction and reperfusion in a simplified manner. As this method cannot fully replicate the complex pathological features of non-experimental ischemic stroke, the generalizability of experimental findings remains limited.

Secondly, most studies use young and healthy animals, as their physiological conditions are more standardized, facilitating experimental consistency. Their strong recovery ability allows researchers to observe a more complete recovery process within a shorter period. In clinic, ischemic stroke predominantly affects middle-aged and elderly individuals, who are often accompanied by chronic conditions such as hypertension and diabetes. These factors significantly influence both the occurrence and functional recovery of ischemic stroke (Lou et al., 2024; Luitse et al., 2012). Therefore, using young animals for research does not fully reflect the pathological characteristics of high-risk stroke populations.

Furthermore, basic studies often use simplified acupuncture protocols to ensure standardization, which differs significantly from clinical acupuncture protocols. To a certain degree, basic studies should gradually align with clinical acupuncture protocols based on animal characteristics to enhance their feasibility for clinical translation. Moreover, existing basic studies primarily focus on cortical ischemic areas, with less emphasis on the more common subcortical ischemic injuries, including the internal capsule, seen in clinical practice (Corbetta et al., 2015). This may be due to the internal capsule being located deep in the brain, with relatively low white matter content in rodent brains, which makes it a significant technical challenge to induce precise lesions in this area (Blasi et al., 2015). Simultaneously, the neural circuits involved are more complex, and research needs to consider the synergistic effects across multiple brain regions, requiring more sophisticated techniques and evaluation methods. This difference may result in incomplete research on the mechanisms of acupuncture, preventing a full research of its comprehensive effects on motor-related brain regions.

In conclusion, the discrepancies between basic and clinical studies may affect the consistency of findings. Future efforts should focus on bridging the two to enhance the clinical translatability of acupuncture mechanism studies.

## 7 Conclusion

In conclusion, this review comprehensively evaluates the mechanisms and clinical characteristics of acupuncture in promoting motor function recovery after ischemic stroke, based on a large body of basic and clinical studies, emphasizing its overall role in functional recovery. We have demonstrated that acupuncture repairs neural structures and reshapes motor function through multiple pathways at various stages of the disease, including restoring energy metabolism, inhibiting neuroinflammation, preventing neuronal apoptosis, promoting neuronal repair and regeneration, and regulating neuronal excitability. Additionally, we explored the key role of different acupuncture protocols in improving motor function and emphasized the necessity of personalized treatment and protocol optimization. Through a deep analysis of these studies, this review provides theoretical support for the application of acupuncture in post-stroke motor function recovery and offers new insights and directions for future research. Further exploration of acupuncture’s potential in motor function repair through modern technologies will expand its application in stroke rehabilitation, providing more practical guidance for clinical treatment.

## Glossary

NSC: Neural stem cell
dMSNs: Direct medium spiny neurons
iMSNs: Indirect medium spiny neurons
ATP: Adenosine Triphosphate
MCAO: Middle cerebral artery occlusion
AMPK: AMP activated protein kinase
I/R: Ischemia–reperfusion
EA: Electroacupuncture
MCT1: Monocarboxylate Transporter 1
ULK1: Unc-51-like kinase 1
FUNDC1: FUN14 domain containing 1
LC3: light chain 3
mTOR: Mammalian target of rapamycin
MMP: Mitochondrial membrane potential
TLR4: Toll-like receptor 4
NF-*κ*B: Nuclear factor kappa B
MyD88: Myeloid differentiation primary response 88
HMGB1: High mobility group box 1
TRAF6: TNF receptor-associated factor 6
IKK: I*κ*B Kinase
JNK: c-Jun N-terminal kinase;
TNF-*α*: tumor necrosis factor-alpha
IL-1*β*: interleukin-1*β*
IL-6: interleukin-6
TNF-*β*: tumor necrosis factor-*β*
JAK: Janus Kinase
STAT: Signal Transducer and Activator of Transcription
IL-4: Interleukin-4
IL-13: Interleukin-13
PPAR*γ*: Peroxisome proliferator-activated receptor *γ*
IL-17: Interleukin-17
IL-21: Interleukin-21
IL-22: Interleukin-22
TGF-*β*: transforming growth factor-beta
IL-10: Interleukin-10
Th17: T helper 17
CXCL1: C-X-C motif chemokine ligand 1
CXCL2: C-X-C motif chemokine ligand 2
IL-17A: interleukin-17A
Bcl-2: B-cell lymphoma 2
Bim: Bcl-2 interacting mediator of cell death
Bad: Bcl-2 antagonist of cell death
Bax: Bcl-2 associated x protein
Apaf-1: Apoptotic protease activating factor 1
ER: Endoplasmic reticulum
Ca^2+^: Calcium ion
UPR: Unfolded protein response
IRE1: Inositol-requiring enzyme 1
PERK: Protein kinase r-like endoplasmic reticulum kinase
ATF6: Activating transcription factor 6
CHOP: C/EBP-homologous protein
MAPK: Mitogen-activated protein kinase
ERK: Extracellular signal-regulated kinase
Akt: Protein kinase B
PI3K: Phosphatidylinositol 3-kinase
PDK1: 3-phosphoinositide-dependent kinase 1
PIP2: Phosphatidylinositol 4,5-bisphosphate
PIP3: Phosphatidylinositol 3,4,5-trisphosphate
GSK3*β*: Glycogen synthase kinase 3 beta
PTEN: Phosphatase and tensin homolog
BDNF: Brain-derived neurotrophic factor
NGF: Nerve growth factor
NT3: Neurotrophin 3
NT4: Neurotrophin 4
CREB: cAMP response element-binding protein
TrkB: Tropomyosin receptor kinase B
TrkA: Tropomyosin receptor kinase A
VEGF: Vascular endothelial growth factor
Nogo-A: Neurite outgrowth inhibitor A
NgR1: Nogo-66 receptor 1
RhoA: Ras homolog gene family member A
p75NTR: p75 neurotrophin receptor
ROCK: Rho kinase
GAP-43: Growth-associated protein 43
NF-200: Neurofilament 200
PirB: Paired immunoglobulin-like receptor B
PSD-95: Postsynaptic density protein 95
SYN: synapsin
SDF-1*α*: Stromal cell-derived factor 1 alpha
Glu: Glutamate
GABA: Gamma-aminobutyric acid
GAD67: Glutamate decarboxylase 67
GABA-T: GABA-transaminase
Cl^−^: Chloride ion
KCC2: Potassium-Chloride co-transporter 2
FMA: Fugl-Meyer Assessment
BI: Barthel Index
EMG: Electromyography
fMRI: Functional magnetic resonance imaging
NDS: Neurological deficit score
NIHSS: National Institutes of Health Stroke Scale
TEAS: Transcutaneous electrical acupuncture stimulation
SRRR: Stroke Recovery and Rehabilitation Roundtable
